# Returning to work: The cancer survivor’s transformational journey of adjustment and coping

**DOI:** 10.3402/qhw.v11.32488

**Published:** 2016-11-15

**Authors:** Antoni Barnard, Loraine Clur, Yvonne Joubert

**Affiliations:** 1 Department of Industrial and Organisational Psychology, University of South Africa, Pretoria, South Africa; 2 Employee Wellness Consultant, Southern Cape, South Africa; 3 Department of Human Resources Management, University of South Africa, Pretoria, South Africa

**Keywords:** Cancer survivor, return to work, adjustment, avoidant coping, emotion-focussed coping, problem-focussed coping, meaning-making, well-being

## Abstract

The aim of this study was to explore cancer survivors’ return to work (RTW) experience with a specific focus on the adjustment and coping process underlying their journey. The study was conducted in the Southern Cape, South Africa, with eight cancer survivors having returned to work following successful treatment of various types of cancer. Unstructured interviews were conducted and data were analysed following the principles of hermeneutic phenomenological reflection and analysis. Four themes emerged, representing the changing adjustment responses and coping during the RTW journey. Participants evolve from being overwhelmed with emotions and applying avoidant coping to seeking understanding and positive affectivity in their attempt to comprehend the reality of their situation. Participants’ external locus of control change to a more active approach and problem-solving orientation, demonstrating a need to take control and responsibility. Ultimately, adjustment and coping become most constructive when cancer survivors resolve to re-assess life and self through meaning-making, resulting in renewed appreciation of life, appropriate life style changes, and regained confidence in their relational role. A process perspective is proposed to facilitate an understanding of, and working with, cancer survivors’ transition through the RTW journey towards optimal coping phases.

In responding to trauma or to any life altering situation, such as a life-threatening medical diagnosis, people inevitably journey a profound intrapersonal change process (Kübler-Ross, 1969; Mayan, Morse, & Eldershaw, 2006). Depending on the magnitude of the trauma experienced, it is accepted that people transition through various phases in adjusting to the new or changed life situation (Parker & Lewis, 1981). Cancer is documented as a traumatic and life-changing event (Smith, Klassen, Coa, & Hannum, 2016) and has developed into a chronic disease (De Jong, Tamminga, De Boer, & Frings-Dresen, 2016; Hoffman, Lent, & Raque-Bogdan, 2013), a reality to which cancer survivors have to adapt.

The body of research, exploring the return to work (RTW) experience of cancer survivors, focusses predominantly on the health and well-being challenges they experience (e.g., Knott et al., 2014; Main, Nowels, Caventer, Etschmaier, & Steiner, 2005), and on the interventions needed to support them when re-entering the work context (e.g., Dewa et al., 2016; Fong, Murphy, Westbrook, & Markle, 2015; Messner & Vera, 2011; Stergiou-Kita et al., 2014). In these studies, RTW is regarded as a period consequent to successful cancer treatment, characterized by various physical and psycho-social challenges; however, research does seem to imply a potential transitory process underlying RTW. A recent study has, for example, focussed on the stage just before re-entering the work context, emphasizing the necessity to determine the work-readiness of the cancer survivor (Stergiou-Kita et al., 2016). Earlier work by Vachon (2001) conceptualizes cancer survivors’ adjustment process as transformative—depicting a perception of the illness as a positive transformation of their lives. The concept of self-transcendence has also been applied to understanding the adjustment and coping responses of cancer survivors (Coward & Khan, 2004). Mayan et al. (2006) describe the process that transform the self of the cancer survivor with the concept of self-reformulation, consisting of attributes describing psychological adjustment behaviour as the ultimate state of healthy adjustment and coping with RTW.

Hoffman et al. (2013) emphasize the need for theoretical models to enhance understanding of cancer survivors’ psychological adjustment as a basis for optimizing their coping and for building well-being interventions. Knott et al. (2014) further highlight the lack of a standard and integrated approach to the RTW rehabilitation process, indicating the need for continued research in understanding the RTW experience. In an effort to contribute to the body of knowledge and to enhance a holistic and integrative understanding of the RTW phenomenon as an adjustment and coping process, the aim of this study was to explore cancer survivors’ experience of their RTW journey.

## Challenges faced in cancer survivors’ RTW experiences

RTW holds significant psycho-social and socio-economic benefits for the cancer survivor (Knott et al., 2014; Main et al., 2005) by enhancing the quality of life (Steiner, Cavender, Main, & Bradley, 2004) and providing a sense of normality and control (Rasmussen & Elverdam, 2008), independence, identity and purpose (Messner & Vera, 2011), social re-connection (Kennedy, Haslam, Munir, & Pryce, 2006), and financial security (Bartley, Sacker, Schoon, Kelly, & Carmona, 2005). Despite the benefits, cancer survivors report numerous challenges in the process of RTW and maintaining employment (Knott et al., 2014; Manicom, 2010; Stergiou-Kita et al., 2014), as the consequences of cancer and its treatment may linger long after treatment (Pryce, Munir, & Haslam, 2007). Challenges differ for each cancer survivor owing to unique work, disease, treatment, and personality-related factors (Hoffman et al., 2013; Spelten, Sprangers, & Verbeek, 2002). They are grouped here as physical, cognitive, and psycho-social challenges.

Treatment-induced physical impairments like fatigue, general weakness, diminished physical capability, impaired cognitive functioning, and loss of confidence are a consistent concern for many cancer survivors (Banning, 2011; Rasmussen & Elverdam, 2008; Shava, 2011; Steiner, Nowels, & Main, 2010; Taskila, De Boer, Van Dijk, & Verbeek, 2011). From a physical perspective, pain remains a challenge cancer survivors have to deal with to a varying extent (Brannon & Feist, 2004), and physical discomfort, such as general fatigue and hot flushes, is a daily reality affecting work capacity (Pullen, 2013). Decline in cancer survivors’ general cognitive functioning, most particularly in language skills, short-term memory, and spatial abilities, has been found (Adler & Page, 2008). Psychologically, cancer survivors have to cope with a sense of loss (Brannon & Feist, 2004), uncertainty, sadness, shame (Messner & Vera, 2011), and the fear of recurrence, resulting in persistent anxiety (Adler & Page, 2008; Lee-Jones, Humphris, Dixon, & Hatcher, 1997; Sarkar et al., 2015). It is not surprising that a number of cancer survivors present with post-traumatic stress disorder as a delayed psychological reaction to the cancer experience (Pat-Horenczyk et al., 2015). Having returned to work, with weakened physical and cognitive capacity, emotional concerns and self-confidence issues, cancer survivors also have to cope with colleagues’ and employers’ limited understanding of the effects of cancer (Munir, Yarker, & McDermott, 2009).

## Coping: a process of psychological adjustment

Coping has long been established as a key concept in adjustment and well-being (Lazarus, 1993) and is defined as cognitive and behavioural attempts to managing potentially conflicting and challenging life demands (Krohne, 2002). In coping with the trauma of cancer diagnosis, its treatment, and the resumption of work life, cancer survivors experience psychological adjustment and transition (Mayan et al., 2006; Pat-Horenczyk et al., 2015). Constructs indicating positive psychological adjustment of cancer survivors such as self-formulation (Mayan et al., 2006), stress-related growth (Park & Fenster, 2004), resilience, personal growth, and mastery (Rowland, 2008) align with the strength-based notion of coping that is fundamental to the positive psychology paradigm (Hoffman et al., 2013). Post-traumatic growth, a construct indicating positive psychological change as a result of coping with trauma (Calhoun & Tedeschi, 2001) has also, for example, recently been explored in a sample of breast cancer survivors (Pat-Horenczyk et al., 2015). Although strength-based coping strategies have thus been emphasized in the context of cancer survivorship, Hoffman et al. (2013) acknowledge impaired well-being and protracted coping in the cancer survivor’s psychological adjustment. As such, one can expect that the cancer survivor’s RTW present with coping orientations and strategies that range the full spectrum of negative–positive adjustment.

Two general types of coping strategies have been associated with cancer, namely problem-focussed and emotion-focussed coping (Folkman & Lazarus, 1980; Hoffman et al., 2013). General coping theory has also identified avoidant coping as a strategy to manage stress (Carver, Scheier, & Weintraub, 1989; Endler & Parker, 1990), and in terms of cancer survivor studies, meaning-making has further been identified as an additional coping strategy (Park, Edmonson, Fenster, & Blank, 2008). Avoidant coping entails denial, and mental and behavioural disengagement (Carver et al., 1989). Emotion-focussed coping is focussed on managing the person’s internal emotive response to a stressor (Krohne, 2002) and includes, for example, acceptance and positive re-appraisal (Carver et al., 1989) and acknowledging, understanding, and expressing emotions (Hoffman et al., 2013). Problem-focussed coping aims at managing the stressor by removing or reducing it (Hoffman et al., 2013) through, for example, active coping, planning, suppression of competing activities, and seeking social support and information (Carver et al., 1989). Whether emotion-focussed or problem-focussed, literature on coping distinguishes between active and avoidant-coping strategies (Rothmann, Jorgensen, & Hill, 2011), providing a potentially useful perspective on aligning avoidant coping as both an affective and a behavioural coping strategy.

Coping has further been distinguished from a personality trait perspective as well as a dynamic process perspective (Rothmann et al., 2011). Understanding the transformative process that is fundamental to cancer survivorship and returning to work (Mayan et al., 2006) is imperative to the cancer survivor and the employer, who has to manage and facilitate the RTW process. A review of coping as an adjustment process should thus include a reflection on relevant meta-theoretical models in this regard. Hoffman et al. (2013) highlight Lent’s (2004) model of restorative well-being as particularly relevant to cancer survivor’s coping process and ultimate psycho-social adjustment. Lent (2004) focusses on how employees restore their subjective well-being through a coping process characterized by an appraisal of the trauma in line with internal coping strategies, affective dispositions, and efficacy beliefs, as well as external psycho-social support resources. Although the model is presented from a process perspective, it rather structures internal and external elements or resources of coping in a self-appraisal framework of being able to adjust in relation to trauma. A more consequential approach to the coping process is proposed by McGrath (2001) and includes a three-phase transition recovery process in adjusting to any kind of trauma, namely circuit-breaking, return of feelings, constructive action, and re-integration. Similarly, Fennell (2012) describes four phases of coping with chronic illness as crisis, stabilization, resolution, and integration. Our hermeneutic reflection on the results in this study was explicitly directed by our interest in cancer survivors’ evolving or changing coping orientation during the RTW journey and our preconception of psychological adjustment to trauma being a process rather than an event or a trait.

## Method

A hermeneutic phenomenological approach was applied to explore cancer survivors’ experience of their RTW journey, specifically in light of adjustment and coping during this process. We were guided by the methodological description of hermeneutic phenomenology of Van Manen (1984, 1990). Van Manen (1984) defines the methodological process as an interaction among four activities: first, the researcher chooses a human phenomenon of interest; second, the phenomenon is to be examined as it is lived; third, the themes that describe the phenomenon, its essence, must be identified; and finally, the researcher engages in the act of understanding the interrelatedness of the phenomenon as a whole (Guimond-Plourde, 2009). To address the phenomenological notion of *openness*, in hermeneutic phenomenology, researcher preconceptions are fundamental to the interpretive process and needs to be explicit in order to clarify the conditions that lead to understanding (Van Manen, 1984; Laverty, 2003). Theoretical preconceptions underlying this study were explicated in the literature review section and researcher introduction follows, to add to the understanding of preconceptions that has influenced this study.

The primary researcher is a female cancer survivor who also volunteers in supporting cancer survivors when they RTW. She is also an experienced interviewer as, in her professional capacity, she has 12 years’ experience in human resources focussing on employee training and counselling whereafter she moved into private consulting with the focus on employment as well as processes related to employee wellness. She found the interviewing process challenging at times, being constantly aware of how participants’ stories reverberated with her own. This required time for self-reflection and debriefing in order to remain empathetic yet open to any new experiences. Sharing an experience with participants enhances the researcher’s ability to enter the research setting and establish rapport with participants (Gill, Stewart, Treasure, & Chadwick, 2008). The second and the third researcher are both academics and professional psychologists (registered with the Health Professions Council of South Africa) with a keen interest in the well-being and adjustment of employees in the workplace. In the hermeneutic phenomenological tradition, and congruent to a social constructionist philosophy of science, the results presented here represent one understanding of the cancer survivor’s RTW process with the intention to contribute to existing and evolving perspectives in this regard.

### Research participants

The participants to the study were recruited from an oncology institution and a cancer support organization in the South Cape and Little Karoo region in South Africa. Purposeful sampling was conducted to seek participants rich in the lived experience (Laverty, 2003; Patton, 2002) of the cancer survivor RTW process. Inclusion criteria constituted having completed treatment consequent to having been diagnosed with cancer, as well as having returned to the workplace. Six participants were recruited via the cancer support organization and the oncology institution. Snowball sampling (Terre Blanche, Durrheim, & Painter, 2006) during interviews with participants led to adding two additional participants. In total, eight cancer survivors participated in the study. The participants fell in various age categories ranging from 28 to 59. Six participants were female and two were male; and five participants were married, two single, and one divorced. Seven of the participants spoke Afrikaans and one’s home language was Xhosa, yet all were well conversant in English. Participants occupied different occupations. Most were office bound and exposed to normal working hours and included a teacher, a financial clerk, a personal assistant, and two managers. The financial clerk as well as two other participants, a car sales lady and a community worker, have the benefit of being able to work flexible work hours. One participant is a pathology officer, which requires manual work such as carrying people on stretchers; however, during his RTW, he was given an administrative position for 6 months. In all these work settings, the participants are required to work with people, although some more than others. Participants were diagnosed with different types of cancer, including breast cancer, lymph node cancer, Hodgkin’s lymphoma, stomach cancer, and cervical cancer. They were all in remission at the commencement of the study. During the course of the study, one participant however received news that her cancer had relapsed and spread. Some participants had invasive surgery such as a mastectomy, together with chemotherapy and radiation. Others only had chemotherapy and/or radiation as part of their treatment.

### Data collection

In hermeneutic phenomenological research, the purpose of the interview as a data-gathering technique is to elicit personal life stories or narratives of lived experience in order to gain a rich and deep understanding of the study phenomenon (Van Manen, 1990). With this purpose in mind, we decided on an unstructured interview format directing all interviews with one open-ended question: “Tell me about your experience when returning to work.” Thereafter the researcher conducting the interviews maintained an in-depth reflective stance by asking participants to elaborate on thoughts and feelings to get a rich description of their experiences (Terre Blanche et al., 2006), with questions such as “How did that make you feel?” or “How did you respond to that?” The interviews were conducted in areas free from distractions and at times and locations that were most suitable for participants. The interviews were all limited to approximately 1 h, and interviews were digitally recorded and consequently transcribed.

### Data analysis

Data analysis was guided by the principles of hermeneutic phenomenological reflection and thematic analysis as proposed by Van Manen (1990). After the interviews were transcribed, each interview transcription was read through repeatedly, concurrent to listening to its recording to ensure accuracy of the transcription and to come to a better overall understanding of the participant’s experience (Van Manen, 1984). Working with an individual interview at a time, sections of text with pertinent meaning in relation to the RTW process were then selected and condensed with meaningful words, labels, or phrases in what Van Manen (1990), p. 92) refers to as “uncovering or isolating thematic aspects.” The selective or highlighting approach (Van Manen, 1990) was used at this stage, by highlighting or underlining significant and revealing phrases in the interview text that would help to understand how the participant experienced the RTW journey. The process of identifying common themes across the transcriptions ensued and formed the basis of developing subsequent common themes across individual interviews. The identification of themes and common themes was done based on a consistent reflection on how the themes related to each other and to the lived RTW experience—within each interview and across interviews. Concurrently, in starting to write the themes, we engaged in composing linguistic transformations resembling meaningful reflections of the RTW journey based on our academic and experiential pre-understanding of coping, adjustment, and cancer survivors’ RTW. Our thematic descriptions evolved through an iterative process of writing and collaborative researcher discussions on interpreting and understanding the themes underlying the RTW process. In these last stages of analysis, our pre-understandings merged to generate a deeper understanding and insight as our research group consisted of researchers who were psychologists and a cancer survivors. As psychologist researchers, our pre-understanding was guided by an explicit interest in the process nature of RTW and the relevant theories of psychological coping and adjustment.

### Ethics

The clinical practice manager at the oncology and cancer support institution acted as the gatekeeper to the study. Permission was obtained from management to put up posters of the study in the reception area and the medical sister was permitted to contact and inform cancer survivors of the study and forward the contact detail of the primary researcher to interested participants. Following contact made by interested participants, the primary researcher set up a discussion during which she re-iterated the aim and nature of the study and explained relevant ethical parameters such as anonymity, confidentiality, and the freedom to withdraw. The study also received ethical clearance from the Institutional Ethics Committee at the University of South Africa and was conducted under the ambit of the ethics policy of UNISA (2013) and the ethical code of the Health Professions Council (Department of Health, 2006).

## Findings

In reflecting on the RTW journey, cancer survivors’ reports demonstrate that their coping and adjustment responses change and evolve throughout the RTW process. It is difficult to ascertain exactly when coping responses change although it seems clear that they do over time change for the better and that participants sometimes find themselves going through that change, yet still having moments of regressing in their coping responses and displaying coping typical to what they applied in more initial adjustment stages. Initially, cancer survivors seem to feel emotionally overwhelmed and respond in irrational and defensive ways to cope in the RTW process, especially because they are faced anew with various physical and emotional challenges in the work context. They however move towards accepting their situation and start speaking of the cancer in factual terms, expressing the need for more information. They also start to make jokes and express hope. In thus becoming more positive in their emotional responses, cancer survivors re-appraise and rephrase their situation in more positive ways and consciously apply tactics to help them physically and socially address the challenges they experience at work. Active and conscious problem-solving tactics increase self-confidence and participants start to reflect on the meaning and value that their lives have derived from being cancer survivors. In this regard, they reflect on their personal growth and value, and they are able to consider how the adjustment process enables them to impact on the lives of others in the work context.

From the data, four themes emerged, reflecting the different and changing emotional adjustment responses and the participants’ coping with the RTW process, namely:

- Avoidant coping with overwhelming emotions
- Seeking understanding and positive affectivity to face reality
- Taking control and responsibility
- Re-assessing life and self through meaning-making

### Avoidant coping with overwhelming emotions

At times, participants seemed to experience the aftermath of the treatment and its physical side effects as a heavy emotional burden linked to a constant fear of premature mortality. Feelings of depression, loneliness, anxiety, and emotional turmoil were expressed as paramount to their daily consciousness and fundamental to the feeling that their situation was emotionally overwhelming. The fear and anxiety of recurrence brought about low moods: “The fragility of your body, it’s actually quite depressing sometimes. For you catch these dips because then you think what’s going on inside of me that I do not know about” (P7); and “I’m very emotional. I have a mild pill because I feel so down and in a deep pit. I hope the pill will help” (P4). In response to struggling with these emotions, participants find they also react more emotionally to situations other than their own, as P8 explains “That is something new happening to me, if something happens to someone else, I also cry. I was not usually like that.” Feeling emotionally overwhelmed, participants struggle to maintain rationality and report feeling unsupported by those closest to them. “My children, they act as if there is nothing wrong. They think I should still be the same” (P2) and “If I picked up the phone and said, listen I need help, I’ll get it, but nothing is offered” (P5). As a result, participants feel dissatisfied and disappointed leading to more irrational questions: “Why me Lord, tell me, I with children, with loved ones. I can’t see Your plan in this” (P4).

In response, participants rely on coping strategies such as avoidance and distraction tactics. As evidence of avoidance, participants deny the need for support in the workplace and speak about the effects of cancer in a detached manner, as if not being much affected by it: “It’s actually my psychological stuff. It’s not work-related and has more to do with the pains and stuff that a guy has. That’s not pain and things that affect my work at all” (P7). Talking thus in the third person about his pain demonstrates detachment and elements of denial. Cancer survivors also relied on distraction tactics to divert their attention from distressing thoughts. Distraction helps them to avoid having to think about the cancer and its consequences and fears of recurrence. Distraction is guided by being amongst other people: “I felt so bad after chemo, but each day I went to work and I always made sure I was among people” (P4); and by keeping busy: “It happens when I am alone, that is why I prefer to be busy. But that is when I am alone then all the emotion will come back” (P8).

### Seeking understanding and positive affectivity to face reality

Cancer survivors started to talk more easily and factually about their cancer experience, not denying the difficulties in having returned to work, or belabouring irrational ideas that this should not have happened to them. The narrative provided by P2 was, for example, mainly focussed on describing her medical situation, challenges, and support in a fairly factual manner:

I first started with radiation. I got ill in September and 6 weeks after surgery I started with radiation because otherwise I would have radiation in December when traffic is worst. My arm started about 3 years after it when I burned it. I cannot pick heavy things up. Sometimes you have to pick stuff up, there’s not always someone to help, but at work I do not pick heavy stuff up there are people to help. If I need help, there are influxes of people. That is good for me.

It seems that cancer survivors seek to better understand their condition and actively explore factual information that would aid in obtaining comprehension. Participant 3, for example, poses a rational question about her current health fears that result from her experience with having had cancer treatment: “After the breast cancer I had my hand operated. It pained and the fingers shake just like that. I wonder if it is because of the treatment? I think I might overtax one arm because I save the other one?” In their attempts to thus speak about their condition and ask questions to better understand, their accepting of reality becomes evident. Acknowledgement of reality of being a cancer survivor and living with the cancer diagnosis and post-treatment consequences coincides with a realization that the cancer impacted them so that they would never be the same as before. The realization does not involve denying the emotional impact as P2 acknowledged her feelings as a reality: “It frustrates me because I ’m used to do things quickly and right. But I say to myself, ah girl remain calm, be calm and then I get it done” and expresses an acceptance of her reality “But it is so, I forget” (P2).

Understanding reality seems to become important and apart from wanting more information themselves about the medical condition and their career alternatives, participants also expressed the importance of information for the employer and co-workers. Coping strategies seem to be emotion-focussed, in that there are attempts at cognitive re-appraisal and the need for acceptance and understanding of the reality. The participants used cognitive re-appraisal by re-evaluating the situation in a more neutral or positive way. Reflecting on being in remission P7 analyses the word to be a negative allusion to still being sick and reappraises “I do not talk about I am in remission. I am healthy, I am healed.” In doing this, they tried to establish positive emotions. Talking about their cancer survivorship seems to spark some hope and led P6 to write a poem:

yet one-day like an eternity passed

Came the message—I was freed

Is there a more perfect joy

To once again taste life

My heart is too small, the feeling too large

There are now life where once was death

Realise that every second of borrowed time

Is a miracle for you and me

Apart from hope, participants also used humour to cope with psychological consequences in relation to body-image, self-esteem, and interpersonal tension. Participant 4 jokes about her hair and her paranoia of getting WhatsApp messages about how she looks:

First time I went to Pick ‘n Pay without hair. There is a small shop outside where I saw a friend and asked her to go with me, because I was so embarrassed at first. I was so relieved, thought now everyone saw me, and then I was fine. I saw no one WhatsApp me, then I looked around, and see no I’m fine.

### Taking control and responsibility

Participants expressed a need for taking action to cope concretely and constructively with the challenges confronting them. Whilst applying a problem-solving orientation to coping, there was however still reliance on emotions and cognitive re-appraisal. The cancer survivors took action by communicating, using support, and managing the challenges.

In trying to explain their illness and gain understanding and assistance, the participants had to be willing to communicate. This was a step they had to take in order to enhance understanding which formed the primary need previously. At times, they experienced difficulties to communicate their cancer-induced challenges for fear of victimization, stigmatization, and feeling ashamed. Moving beyond this fear, participants started saying that being open and honest in communicating the challenges they experienced improved their chances of acceptance and understanding. According to P1 “not everyone, just those on my shift know” that he had cancer, revealing that it was an embarrassment to him and he did not feel comfortable in letting everyone at work know about his illness. The story told by P5, however, shows how she has changed in this regard: “I am very straightforward, open and honest. As I get hot flushes, I’ll say: ‘Excuse me, I am now very hot’ and then cool myself down.”

Several narratives displayed active strategies employed by the participants to manage the challenges they faced. In order to address her forgetfulness, P5, for example, says that “To remember, I write everything down. I don’t, I will forget,” and P2 consciously heeds her physical needs: “I get very tired. I get so tired that I become nauseous. My body says sit down otherwise you are going to vomit. If I rest 15 minutes then it gets better, if I just sit” and “Writing on the blackboard is hard, but I made a bench for myself to stand on to make it easier.”

With a stronger focus on problem-solving as a coping strategy, cancer survivors continue to rely on emotion-focussed strategies and cognitive re-appraisal, albeit in a manner that continues to be more constructive. As opposed to feeling emotionally overwhelmed and despondent, cancer survivors start to display a stronger internal locus of control, taking responsibility for their reality: “It depends on myself to stay motivated. Am I always going to be emotionally down or will I lift myself out of it?” (P1). Cognitive re-appraisal re-appears in the form of enhanced self-confidence and determination to take control as is evident in the words of P8: “You see when I came back people said: ‘You are not supposed to wash patients’, ‘You are not supposed to do this’, ‘You are not supposed to do that’. I said: ‘I am going to do that’.” Problem-solving is furthermore exemplified in the setting of goals to be strived to and worked for: “I’m going to work at GC next year…” (P4) and “I’m carrying on with my studies. I want to do everything as before I was diagnosed with cancer” (P8). “I’m going to make the best of it” and “I will not lie down” (P1).

### Re-assessing life and self through meaning-making

Participants’ narratives at some stage reflected going beyond acceptance, understanding, and a constructive and active coping orientation. Here cancer survivors focus on incorporating *meaning* from their cancer experience into their lives through re-assessing their personal values and life orientation, their relationship investments, and their lifestyle priorities.

Several participants commenced reflecting on the positive effect cancer had in their lives and reflected on the positive value and the meaning of being a cancer survivor. Some participants, for example, experienced a positive change in themselves and seem to have developed a stronger appreciation of life in general:

Emotionally or if you look personality-wise, I am less self-centred than before. I have more compassion with other people. I have more patience than before. I experience pretty things, nice things, more intensely that I perhaps would have done previously. I am more grateful, uhm and I am stronger emotionally, especially with what I do. (P5)

The intrapersonal change experience is demonstrated in a changed attitude or orientation to life in general as noted by P4: “A person is always precise in your work. I was always precise, but now, if something is not done tomorrow is another day. I am not so worried about everything.”

The cancer survivors recognize and enjoy the change that had occurred in them resulting in a different way of investing in their relationships. Relationships with others seem to gain a deeper level of understanding and appreciation: “But underneath there is a thing in our family, a very much greater understanding for the time that we spend together” (P7). Cancer survivors reflect on the meaningfulness of having been diagnosed with cancer, surviving it, and in their RTW journey they become aware of being able to impress on other people important life lessons: “They say, I can’t use that machine, I can’t do this, I can’t do that. I end up pushing my top up and showing them my scar, my big scar. So I told them if a person like me can wash a patient, can push a bed around, I think you can also do it? That was the end of the story, they just carried on” (P8).

The re-assessment of values and priorities has not only led to an awareness of changed life orientation and spending more time with family, but participants also note making conscious and constructive lifestyle changes as is evident in the words of P7:

I know if I am going to boost my body with the right food I can definitely recover the damage that is done within me. I can recover because that’s how our bodies work. I have to give it enough fuel. Do you enjoy everything as always and you overeat on steaks and ice cream and such or do you say no, I’ve got a second chance and use it right.

## Discussion

The analytic process confirmed the physical, cognitive, and psycho-social challenges cancer survivors experience during RTW as identified in previous research (Knott et al., 2014; Main et al., 2005). The fluctuations in coping with these challenges however provided a new insight in that although the challenges remain, the cancer survivors’ coping orientation, their emotive responses, and their coping behaviour changes as the RTW journey unfolds. The seminal writing on coping research by Lazarus (1993) highlights the importance of personal appraisal in understanding coping behaviour. The individual’s appraisal of the stressor directly affects the chosen coping strategy and emotional responses serve as a barometer of the level of adjustment attained (Lazarus, 1993). Changes in cancer survivors’ orientation to cancer in their RTW journey and changes in their consequent emotional responses, therefore, serve as an indication of a transformational coping and adjustment process. The cancer survivors appraised their situation differently at different times in the RTW journey and concurrently reported changing coping strategies to protect and optimize their well-being.

**Figure 1:**
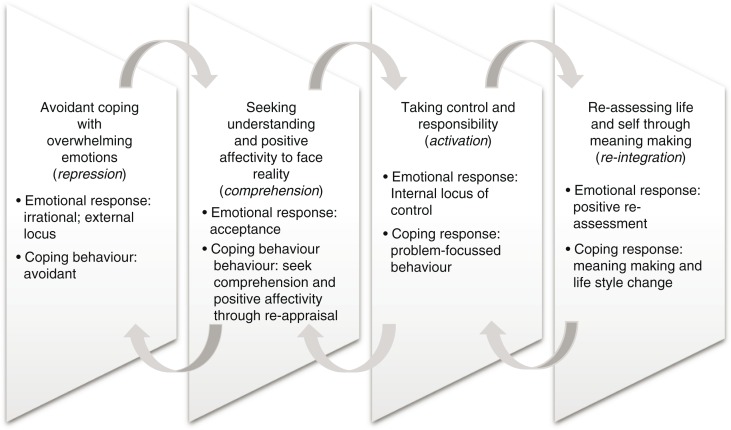
Coping with RTW: transformational adjustment through four phases.

Overall the four themes combine to structure the essence of the RTW journey as a process of adjustment and personal transformation, consisting of four successive yet interrelated coping phases, depicted in Figure 1. The basic assumption of our phased understanding of the RTW journey holds that despite challenges remaining, the perception of these challenges, the way of coping with them, and the employees’ expectations in the work context change throughout the RTW process. Similar to Fennell (2012), we prefer using the term “phase” to “stage,” because stage implies a progression that only goes forward, whereas phase implies that a person could go back to an earlier phase when new challenges occur. The changing coping orientation seems to be an iterative adjustment process because cancer survivors’ expression of coping strategies varies from time to time demonstrating forward yet returning movements between the phases identified in the themes. The avoidant, emotion-focussed, problem-focussed, and meaning-making coping strategies associated with trauma in general (Carver et al., 1989), and cancer in particular (Folkman & Lazarus, 1980; Hoffman et al., 2013), are integrated in the adjustment process proposed here. The adjustment process of the RTW journey is depicted in the movement from avoidant coping with overwhelming emotions to seeking understanding and positive affectivity to face reality, then taking control and responsibility, and finally a re-assessment of the self and of life through meaning-making.

The adjustment in the RTW process represents an evolvement of emotional and behavioural coping responses that can be aligned to the phases of chronic illness (Fennell, 2012) and phases in the process of recovering from trauma (McGrath, 2001). The alignment of the study themes with the models of Fennell (2012) and McGrath (2001) is discussed later and led us to uniquely label the four phases in our process perspective to cancer survivors’ RTW coping and adjustment (Table I).

**Table I:** Alignment of study themes and adjustment models.

Themes	Fennell (2012)	McGrath (2001)	Phases in RTW process
Avoidant coping with overwhelming emotions	Crisis	Circuit-breaking	Repression
Seeking understanding and positive affectivity to face reality	Stabilization	Return of feelings	Comprehension
Taking control and responsibility		Constructive action	Activation
Personal growth through meaning-making	Resolution	Re-integration	Re-integration
	Integration		

RTW, return to work.

In the initial phase, cancer survivors experience RTW challenges as emotionally overwhelming and respond with an avoidant-coping orientation that leans towards being irrational and representative of an external locus of control. This phase is similar to the first phase of circuit-breaking in the trauma recovery process described by McGrath (2001) which reflects overwhelming emotions, such as shock, resulting in numbness. We found avoidant-coping strategies such as denial, detachment, and distraction to be predominant in this phase, representing an avoidance of reality. As opposed to suppression which is a conscious defence, repression as an unconscious defence (Erdelyi, 1990) seems to be appropriate to describe cancer survivors’ avoidant response in this phase. Repression represents how cancer survivors apply mental defence mechanisms in response to overwhelming emotions to deny, repress, or negate unpleasant or hurtful realities in protection of the self. In general coping literature, avoidant-coping strategies typically include denial, mental, and behavioural disengagement (Carver et al., 1989; Rothmann et al., 2011). Some regard avoidant-coping strategies as a psychological risk and unsuitable to constructively address stressful life events (Holahan & Moos, 1986). Denial has however been considered a useful coping mechanism (Carver et al., 1989; Lazarus, 1993), yet more healthy forms of minimizing distress such as positive affectivity and cognitive re-appraisal evolve in the process of recovering from trauma (McGrath, 2001).

Moving from avoidance, cancer survivors express a need for understanding and positive affectivity in order to face reality. Their expression of hope and use of humour as well as open engagement with cancer information is similar to the stabilization phase identified in Fennell’s (2012) model of chronic illness, wherein the need is to structure life and perceptions in line with the trauma. Other cancer survivor studies also highlight the psychological containment effected through the use of humour (Labrecque, Coutu, Durand, Fassier, & Loisel, 2016; Romero & Cruthirds, 2006). In our study, cancer survivors display an acceptance of their relationship with cancer and express a need for more information and knowledge about being a cancer survivor in the RTW process. They are able to talk about their journey with cancer in a more rational and objective, even factual manner. Acceptance of cancer survivor identity starts pointing to an improved psychological adjustment, whereas a victim identity denotes poorer well-being (Park, 2013; Smith et al., 2016). Participants in this study now also start to employ rational-emotive coping strategies, balancing a re-appraisal of negatives with the more constructive emotional-focussed tactics such as humour and hope. According to Lazarus (1993), emotion-focussed coping involves the re-appraisal of the stressor in more positive ways to mitigate the effect of the stressor. Positive emotions broaden a person’s thoughts and actions, helping to build personal resources (Fredrickson, 2001). Emotion-focussed coping in this phase is therefore rather directed towards changing one’s orientation and emotional response to the cancer as opposed to attempting to change the situation itself (Krohne, 2002; Manne, 2007). Acceptance of reality seems to be fundamental to cancer survivors’ comprehension of their situation on both cognitive (understanding) and emotional (re-appraisal) levels.

Cancer survivors changing their focus from emotion-focussed coping to being focussed on actively solving practical problems evidences another turn in the RTW adjustment process. Various instances of problem-focussed coping in the RTW process discovered here reflect practical ways to manage or reduce the impact of the stressor as noted by Hoffman et al. (2013) and Lazarus and Folkman (1984). Application of strategies focussed on problem-solving concur with a stronger internal locus of control and accepting responsibility for going forward which is one of the coping strategies typical to coping with stress in general (Lazarus, 1993). Such a problem-focussed perspective denotes activation of resources and is similar to the constructive action phase identified by McGrath (2001) wherein people respond to trauma by taking action to restore a sense of control and normalcy.

Advanced coping strategies and adjustment responses in our study were demonstrated in the personal learning, growth, and meaning-making attempts by cancer survivors. Psychological meaningfulness and meaning-based coping has long been highlighted as essential to employee-subjective well-being (Rothmann & Hamukang’andu, 2013), a resilient coping orientation (Antonovsky, 1987), and specifically to cancer patients’ constructive coping (Lethborg, Aranda, Bloch, & Kissane, 2006). As in the meaning-making model proposed by Park (2013), meaning-making in this study is derived at through the increasing awareness of a changed life orientation based on re-appraised personal values and priorities. Meaning was furthermore evident in a different way of qualitatively investing in relationships and gaining confidence in also impacting the lives of others constructively. Lifestyle changes are done from the vantage point of accepting accountability for personal health. Meaning-making as a coping strategy described in this study shows distinct similarities with the idea of stress-related growth which includes positive relationship changes, enhanced personal resources and coping skills, changed worldviews, and healthy lifestyle behaviours (Park, 2009). Cancer survivors’ behavioural coping strategies thus integrate a constructive emotion-focussed style with problem-focussed tactics, as well as meaning-making strategies. According to Park (2013), in low-control situations, such as serious illness, meaning-making is most beneficial to psychological adjustment, yet meaning-making may still include emotion-focussed and problem-focussed coping strategies. The task of meaning-making in recovering from trauma is evident in McGrath’s (2001) fourth phase of re-integration, where learning and growth takes place, and the meaning of the trauma is incorporated into one’s life. Fennell (2012) addresses meaning-making in her model’s resolution and integration phases. The resolution phase emphasizes the need to develop a new identity and find a meaningful philosophy to live by. In the following integration phase, the main task is to make life and work style adjustments, to continue to find ways to re-integrate back into socio-economic life, and to position the illness within a larger philosophical or spiritual framework. Total re-integration is described as arriving at a new complete experience of life in which illness forms only a part (Fennell, 2012).

## Conclusion

The aim of this study was to explore cancer survivors’ experience of their RTW journey in an attempt to better understand the coping and adjustment of cancer survivors in the process. The data elicited four process-related themes highlighting the emotional responses and coping strategies particular to each phase in the RTW process. The context-specific, qualitative, and hermeneutic nature of the study hold limitations in terms of transferability that need to be kept in mind when considering the results. The homogeneity in terms of cancer survivorship however lent us a focus on the process of adjustment and coping, providing a useful understanding of the RTW journey. To address rigour, the data analysis and verification process entailed a transparent declaration of researcher intent and preconceptions, a detailed account of our hermeneutic lenses, as well as iterative rounds of data analysis conducted in collaboration with three researchers.

Understanding RTW from a process perspective to coping may facilitate focussed yet integrated support to guide the transition of the cancer survivor through all the phases in order to reach the final and optimal adjustment phase. An understanding of the needs of cancer employees, as they transition through their RTW journey, could also facilitate self-awareness and self-management in the adjustment process. Cancer employees as well as employers have a responsibility to work towards a successful RTW process to ensure optimal adjustment, re-integration, and productivity of the cancer survivor in the work context. A process orientation to RTW, as the one suggested here, may further establish grounds for a phased support approach and a clear RTW plan that permeates the adjustment needs and coping strategies cancer survivors experience throughout the RTW journey. The findings could also facilitate assessment of cancer survivors’ coping efficacy at any given time and identify support needs to progress to more efficient coping phases. Finally, we believe the process perspective to RTW adjustment potentially provides a unique and integrative way to counsellors, employers, and other role players of understanding and working with cancer survivors coming back to work. Yet, we recommend further research in this regard to confirm the potential value of such an approach.
